# Instrumental Activities of Daily Living (I-ADL) trigger an urgent request for nursing home admission

**DOI:** 10.1186/0778-7367-70-2

**Published:** 2012-01-03

**Authors:** Gilberte Van Rensbergen, Jozef Pacolet

**Affiliations:** 1 Catholic University of Leuven, Belgium; 2 Social and Economic Policy Unit, Higher Insitute of Labour Studies, Catholic University of Leuven, Belgium

## Abstract

**Objective:**

Although disabled elderly people mostly prefer to receive care at home or in other community settings, many of them reside in nursing homes. That is why several researchers have tried to identify predictors of institutionalisation. Various different dependency factors seem to explain the request for residential care. The aim of this study is to discover the most important factor triggering an urgent request for nursing home admission.

**Methods:**

On the basis of social field research, we analysed the profiles and motives of an admission cohort of 125 elderly (31 men and 94 women) who were admitted to four nursing homes in Antwerp (Belgium) between January 2000 and April 2001. The study used data of the 'intake conversation', performed by an experienced social worker of the nursing home, subsequent to the request for nursing home admission.

Gender, age, Katz category, marital status, disease, living conditions, Personal and Instrumental Activities of Daily Living (P-ADL and I-ADL) were the independent variables.

The variable 'time span' was introduced as dependent variable. This is the time between the onset of dependency and the request for institutionalisation. Nursing home carers have classified this time span in three periods: < 3, 3-12, and > 12 months. The statistical analysis focused on the characteristics of the two extremes, namely the earliest versus the latest applicants (n = 74). This was the best strategy to go about investigating the issue due to the vagueness and uncertain status of the data in the midrange.

**Results:**

The applicants had an average age of 83 years. 31% of the elderly were defined as functioning good (needing assistance from another person in no to maximum two ADLs - washing and dressing) and 69% were classified as ill functioning (needing assistance in minimum three ADLs). Women were more likely to be widowed (83%) and to live alone and isolated (55%) and they had a lower degree of dependency (both P-ADL and I-ADL) when entering institutions. Of the women, 57% had a mental illness, compared with 48% of the men. Of the applicants, 34% were unwilling or unable to start home care and applied for an urgent request (within the first 3 months); 41% tried home care for a time and 26% applied after one year of home care.

The stepwise logistic regression analysis identified I-ADL as the decisive factor explaining the difference in 'decision speed' towards institutionalisation. An increase of one unit on the I-ADL score increased the chance of a request within the first three months by 63% (95%CI: 19 to135%, p = 0.006).

**Conclusions:**

The only factor related to an urgent request for nursing home admission seems to be the I-ADL score. These results have important implications for targeting sheltered housing and further extension of home care services to postpone or prevent institutionalisation.

## Background

### Nursing home care versus home care

Care of the elderly in Belgium ranges from relatively high standards of residential care (long-term nursing home care) to community or home care (consisting of informal care, professional home help and district nursing).

Due to the persistent ageing of the population, both types of care have developed together in the past two decades, a trend which may also be observed in other European countries. There is no real substitution, but rather joint development [1,2].

In Belgium, nursing home admission is in principle free for everyone who applies. There are no limits in terms of distance, no social and economic thresholds, and no health restrictions [3]. In this 'merged system' of rest home and nursing home the elderly can move between different levels of care without having to leave the facility (no transfers necessary)[4].

Nowadays, the view on care is changing, with a trend towards promoting non-institutional concepts. In current practice, initiatives are presented considering the needs and wishes of elderly people with respect to care, housing and other aspects of wellbeing [5]. Elderly people dream of living independently but free of troubles and concerns. They are searching for a feeling of safety and security, social contacts and in particular a confidant who is permanently available to solve the problems they cannot solve themselves.

### The reason for nursing home admission

The reason for nursing home admission is often a combination of factors [6]. Jette et al. examined the predictive power of 11 predisposing, 7 enabling and 18 need factors [7]. In the 1990s/2000s there have been a large number of studies that have examined predictors of nursing home placement. The most commonly identified personal risk factors include advanced age, gender (women), levels of Personal Activities of Daily Living (P-ADL) and Instrumental Activities of Daily Living (I-ADL) (especially restricted outside mobility), mental impairment, living alone, dissatisfaction with one's living situation and the presence of specific medical conditions [3,8-13].

Elderly people first require help for household activities and only later for personal care [14]. The importance of the presence of informal care and/or formal home help, supporting the I-ADL needs, is demonstrated by several authors [15-18].

### What finally triggers an urgent decision to institutionalise people?

In general it is assumed that the majority of elderly people would prefer to stay at home as long as possible and choose residential care as final option. But is there really a free choice? "Many applicants probably enter nursing homes for reasons that are not fully compelling"[19]. "It is very likely that a substantial number of elderly people currently requesting nursing home admission, can be helped by home help services that delay (or prevent) nursing home placement" [20].

The aim of this pilot study is to investigate which is the most important factor triggering an urgent request for nursing home admission.

## Method

### Description of the study

### Population

Between January 2000 and April 2001, an admission cohort (the last 30 to 35 new entrants) (n = 125; 31 men, 94 women) at four nursing homes in Antwerp was screened by the social service of the nursing home via face-to-face interviews. After the request for nursing home admission, it is customary to visit the future resident at home or in hospital to evaluate her/his functioning and living arrangements. Proxy interviews were used when direct interviews were impossible because of mental impairment or poor health. An experienced social worker screened in detail the applicants using a structured and reliable questionnaire. From this 'intake conversation', sociodemographical data and scales of need of each participant were collected. A concise medical reason for admission (i.e. stroke, hip fracture, dementia) was also assessed. The compiled database was treated in accordance with ethical guidelines of complete confidentiality and analysed with the approval of the ethics committee.

### Data collection of the independent variables (categories and scores)

The independent variables were: gender, age, the official Belgian Katz score (an adapted Katz scale), marital status (co-resident versus living alone), a measure of Personal and Instrumental Activities of Daily Living (P-ADL and I-ADL), the mental or physical nature of the disease and positive or negative living conditions (including a combination of several factors).

The variable 'living conditions' had to be dichotomised because a lot of different overlapping responses were given i.e. illness or the loss of a co-resident or principal carer, feelings of insecurity, social isolation, recent hospitalisation, availability of professional home help, inappropriate housing conditions (too extensive living area, large garden) etc.

A positive living condition included living together with others, or living in the proximity of helpful people (protected living). A negative living condition is the opposite.

Residents that are defined as 'in need of care' are dependent on another person for P-ADL needs, measured by means of an adapted Katz scale. The six P-ADL items, used to classify residents' physical functional status, are: washing (personal hygiene), dressing, mobility, toilet use, incontinence and feeding. The mental items include disorientation in time and place. A score (1 to 5) was given for each item and those were summarised.

The adapted Katz scale allows the classification of each person distinguishing four main categories of ability. Residents of Katz category O do not need assistance with any ADL; category A need assistance in two ADLs (washing and dressing); category B require assistance in three and category C in more than three ADLs. This classification, assessed by a physician, a social worker or a nurse, is comparable with the international criteria [20]. We used this classification to define good functioning (category O or A: needing assistance from another person in no more than two ADLs) and ill functioning (category B or C: needing assistance in minimum three ADLs).

To categorise I-ADL needs, we used a simplified version of five out of the original 18 items from a 'General List of Household Activities'. These five activities, considered to be relevant by the social services, are: housecleaning, cooking, mobility outside, laundry/ironing and administration.

### Data collection of the dependent variable

**Figure 1:**
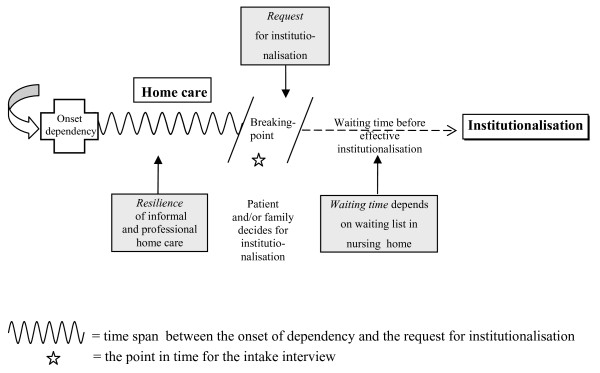
**Time span between the onset of dependency and the request for institutionalisation**.

We asked the social workers to introduce the variable 'time span'. This is the time span between the onset of dependency and the request for institutionalisation (Figure 1).

Onset of dependency is defined as the moment a person first mentions not being able to function alone without help from another person. The degree of urgency of a request can indirectly be objectified by measuring the time span between this onset of dependency and the request for institutionalisation. Nursing home carers have classified this time span in three periods: less than three months, from three to twelve months and more than twelve months. These categories reflect the possibility and availability for initiating (3-12 months) or not initiating (< 3 months), or for maintaining (> 12 months) home care. It is an indication of the 'resilience' period of home care and depends among others on the living conditions, relationships and social support, the availability of sufficient and timely home help services.

In this paper we do not focus on the "waiting time" which is the amount of time between the application for admission and the institutionalisation (Figure 1).

### Data analysis

Searching the profile of those requesting an urgent or late request, we tested the significance of the different variables. The scores for P-ADL and I-ADL were '0' for independency, '1' for a low level and '2' for a high level of dependency, which leads to possible maximum scores of 12 for P-ADL and 10 for I-ADL. These ordinal data were processed like continuous variables (addition sums). After calculating the totals, we used a Kruskal-Wallis test.

Pathology was coded either as a physical disease or as a mental illness. Living conditions were coded as a positive or as a negative environment; (dichotomous variable after the use of dummy variables). We used the chi-square test for these discrete data.

To identify the statistically most significant variable, triggering an urgent request for admission, we performed a stepwise logistic regression with 'time of request' as a dependent variable. This variable was dichotomised as 0 when the time was less than three months and 1 when it was more than twelve months. The statistical analysis focussed on the characteristics of the two extremes, namely those with a short resilience period (less than 3 months) versus those with the longest resilience period (n = 74). The in-between category with a resilience period of between three and twelve months was excluded from the analysis because of recall issues: elderly people are unable to remember the exact date of the onset of the need for care. Estimates made during the first three months and more than a year after onset of the need for care yield the most precise results.

## Results

### Descriptive characteristics of the population

In the sample of 125 cases, comprising 31 men (25%) and 94 women (75%), the average age was 83 years, with a median of 84 and a Standard Deviation of 7. Men and women had similar age profiles. The minimum and maximum age upon admission was 52 and 95 years respectively. The characteristics of the population are shown in Table 1.

**Table 1:** Characteristics of a sample (n = 125) of new entrants in four nursing homes (%)

	Men (n = 31)	Women (n = 94)
**Dependency scale (Katz)**		
Category O/A	8 (26%)	30 (32%)
Category B/C	23 (74%)	64 (68%)
		
**Marital status**		
Widower/widow	14 (45%)	78 (83%)
Married	17 (55%)	16 (17%)
		
**Living conditions**		
Living alone (negative living condition)	11 (36%)	52 (55%)
Living protected (positive living condition)	2 (6%)	12 (13%)
Living together (positive living condition)	18 (58%)	30 (32%)
		
**Disease**		
Somatic disease	16 (52%)	40 (43%)
Mental disease	15 (48%)	54 (57%)
		
**Total**	**31 (100%)**	**94 (100%)**
		
**I-ADL (dependent from others)**		
Cleaning	29 (94%)	80 (85%)
Cooking	28 (90%)	74 (79%)
Mobility outside	21 (68%)	62 (66%)
Laundry/ironing	28 (90%)	70 (74%)
Administration	19 (61%)	66 (70%)
		
**P-ADL (dependent from others)**		
Washing	17 (55%)	46 (49%)
Dressing	17 (55%)	44 (47%)
Mobility	12 (39%)	31 (33%)
Using the toilet	12 (39%)	34 (36%)
Incontinence	9 (29%)	17 (18%)
Eating	4 (13%)	9 (10%)

- For the dependency category, marital status, living conditions and disease, the percentage is expressed relative to the size of the specific population.

- For the I-ADL and P-ADL scales, several items can be indicated simultaneously. For instance, 9 out of 10 men are dependent on help from others for cleaning, cooking and laundry.

- Positive living conditions included living together with others, or living in the proximity of helpful people (protected living). Negative living conditions are the opposite.

The distribution of elderly people (men and women) on the Katz scale was as follows: good functioning: 31% (category O: 14%, category A: 17%), ill functioning: 69% (category B: 30% and category C: 39%). When entering the nursing homes, the prevalence of good functioning was higher in women (32%) compared to men (26%).

Fifty percent of the men and 36% of the women were categorised as highly dependent (category C). Women were more likely to be widowed (83%) and to live alone and isolated (55%) ('isolated' as opposed to 'protected'). The need for I-ADL support precedes the need for help in P-ADL. Even among subjects with good functioning (category O or A on the Katz scale) there was a dependency for I-ADL. The tasks where the dependency occurred first were cleaning followed by cooking. For men, doing laundry and ironing are also problematic tasks. Administrative tasks and mobility seem to be possible for a long time.

As expected, the scores for P-ADL show the hierarchical order of functional physical deficits as determined by Katz. Washing and dressing score the highest, eating and incontinence the lowest. If the scores for mobility and toilet use were to switch, the cause could be found in pathology. Persons suffering from dementia remain mobile longer.

Of the 94 women, 57% has a mental illness, compared with 48% of the men. Forty-nine per cent suffer from dementia, and 10% of the men and 15% of the women (especially the younger ones) suffer from depression.

### Time span between the onset of dependency and the request for institutionalisation

When the request for admission occurs within 3 months after the onset of dependency (e.g. during the patient's stay in the hospital) this implies that people are not *willing *(e.g. because the co-resident is overburdened) or not *able *(e.g. because of the unavailability of sufficient professional home care services at that moment) to start home care.

In our sample, 34% of the respondents (men 35.5%; women 33%) were unwilling or unable to start home care, implying an urgent request. Forty-one per cent tried home care for a time and 26% had effectively used home care for more than one year (Table 2).

**Table 2:** Time span between the onset of dependency and the request for institutionalisation, according to gender and degree of dependency

Time span between the onset of dependency and the request for institutionalisation	Men (n = 31)			Women (n = 94)		
	**O/A**	**B/C**	**Total %**	**O/A**	**B/C**	**Total %**

≤ 3 months	6 (**75 **%)	5 (21,7%)	11 (35,5%)	14 (**46,7 **%)	17 (26,6%)	31 (33,0%)
3 to 12 months	1 (12,5%)	11 (47,8%)	12 (38,7%)	11 (36,7%)	28 (43,8%)	39 (41,5%)
≥ 12 months	1 (12,5%)	7 (30,4%)	8 (25,8%	5 (16,7%)	19 (29,7%)	24 (25,5%)

**Total**	**8** **(100%)**	**23** **(100%)**	**31 (100%)**	**30** **(100%)**	**64** **(100%)**	**94 (100%)**

There is no difference in timing between the male and female respondents. Mostly they apply within 3 to 12 months (38.7% of the men and 41.5% of the women). Yet, there is a paradox. The *least dependent *elderly (category O/A) were also among those making the *earliest request *(≤ 3 months) for admission (men 75%, women 46.7%).

### I-ADL trigger an urgent request for institutionalisation

All the major variables had a statistically significant impact on the time span. The results showed that I-ADL (*χ^2 ^= 9.76; p < 0.01*) and P-ADL (*χ^2 ^= 9.72; p < 0.01*) as well as disease (*χ^2 ^= 4.57; p < 0.05*) and living conditions (*χ^2 ^= 5.65; p < 0.05*) are important in their own right. Looking for the most significant variable, we performed a stepwise logistic regression on 'time span between the onset of dependency and the request for institutionalisation' (≤ 3 months versus ≥ 12 months) as dependent variable (Table 3). The analysis identifies the Instrumental Activities of Daily Living ( I-ADL) as the most important factor explaining the difference in 'decision speed' for institutionalisation. An increase of one unit on the I-ADL score increases a request within the first three months by 63% (95% confidence interval: 19 to 135% p = 0.006). None of the other possible covariates that were tested reached the level of significance.

**Table 3:** Results of the logistic regression on the determinants of the time span between onset of dependency and request for institutionalisation (t-value between brackets) (n = 74)

	Time span	Time span	Time span	Time span	Time span	Time span
**Independent variables**						
						
GENDER	0.0496 (0.0740)					
AGE	-0.0206 (-0.4882)	-0.0207 (-0.4965)				
KATZ	-0.1889 (-0.2385)	-0.1950 (-0.2474)	-0.1946 (-0.2488)			
MARITAL STATUS	0.0492 (0.0674)					
I-ADL	0.2960 (1.3827)	0.2909 (1.3958)	0.3007 (1.4477)	0.3088 (1.5104)	0.3844 (1.9430)	0.5067 (2.8716)
P-ADL	0.0806 (0.8053)	0.0845 (0.9240)	0.0837 (0.9192)	0.0941 (1.1570)	0.0794 (1.0046)	
DISEASE	-0.7245 (-1.2159)	-0.7145 (-1.2138)	-0.7041 (-1.2027)	-0.7134 (-1.2202)		
LIVING CONDITIONS	0.0136 (0.0087)					

## Discussion

### Limitations of the study

Due to the extensive social screening, only four nursing homes were included in our study, which might not be representative for the elderly population at large. Yet, our research supports the general observation that maintaining the house often constitutes the biggest stumbling block. Our sample size is limited: 125 of which 74 for the logistic regression.

The statistical analyses focussed on the 74 cases at the extremes of the scale, namely the earliest versus the latest applicants. Due to worries about the vagueness and ambiguity of the data in the midrange, the in-between category (3-12 months) was excluded from the analysis. This was the best strategy in light of the data to investigate the current question.

The time spans between onset of a need for care and a request in our sample showed a skewed distribution. Most people put in a request within the first six months. In addition to that, estimates of the onset of a need in the midrange of that time scale were less certain than at either end of the scale. Elderly people or their families are unable to give the precise week/month when the need for care actually began and in general, their situation deteriorates slowly. However people that cannot cope put in a request within the first 3 months and people that can cope manage for at least a year and beyond. The strategy therefore employed here was to use the cut-off point of 3 months (used by the professionals) and 12 months and focus the statistical analyses on these extreme points.

The selection of the independent variables was perhaps not the most appropriate for the logistic regression, but it concerns standard parameters. The interaction between some variables is inherent to this kind of research. Married people have more chance of I-ADL help since they have someone to do the chores for them.

The study focussed on observed data as the needs are assessed by professionals according to standardised scales, rather than on subjective data. Over the past few years, practical experience has shown that in addition to these objective needs, subjective (social-emotional) needs play an increasingly important role. For instance, dependency in terms of outside mobility cannot be entirely resolved by means of a shopping service. Once people are no longer able to get about on their own, a great many social activities are no longer possible which can lead to extreme isolation.

Minor local variations in the scope of home care support can sometimes determine whether an elderly person can stay at home or not. Mostly this involves organised voluntary work, e.g. 'granny sitting' (day and night).

### I-ADL is the most prominent factor for the urgency of a request for admission

An urgent request seems to be mainly determined by deficits in household activities. It signifies that the score on the Katz scale is not the most important reason for admission to institutional care although physical or mental shortcomings are the key criteria in legal terms. This is the explanation of the paradox in Table 2 which showed that the elderly without or with limited P-ADL limitations (category O/A) were also among those making the earliest requests. Crucial are deficiencies in performing Instrumental Activities of Daily Living.

It is likely that there is confusion between I-ADL and living conditions but they are not entirely similar. The one is on the demand side, the other on the supply side. I-ADL is the objective need assessment for specific household activities. The broader living conditions point to the presence or absence of a positive microclimate (including housing, protected living which gives some feeling of security, supervision and concrete forms of P-ADL or I-ADL help).

Also living conditions and marital status are not covering the same. This is shown in Table 1: only 17% of the women was married whereas the living conditions of 45% of them were qualified as positive. Some authors indicate that improved P-ADL (self-care) does not guarantee a diminishing need for I-ADL support. 'The need for help with the Instrumental Activities of Daily Living did not change, even with better physical conditions' [21]. A population-based survey among the elderly in Belgium has shown that the number of people aged over 65 with severe P-ADL problems fell from 25% in 1966 to 16% in 2001. Problems with I-ADL declined too, from 28% in 1966 to 14% in 2001 but there was no parallel decline in the 'demand' for professional I-ADL supports [22].

### Professional and informal home care do not currently adequately meet the I-ADL needs

Although community care was promoted during the last 20 years, there was a further increase in institutional care. This might be the result of a caring gap in the provision of I-ADL support, both informal and professional care. As our study shows, the greater the I-ADL need, the sooner people move into an institution, we can suggest that the level of home help coverage is not related to home help needs.

Waiting list times for professional home help can be as high as half a year. Not the payment for domestic tasks is a problem, rather a probable shortage of personnel. "The strain will be less on the financial side than on the shortage of services and people willing to render the care required" [23].

Belgian research revealed that 17% of the current O- and A- residents in nursing homes could have stayed at home, with support of the community [24]. "A sizeable proportion of those admitted to nursing homes could be kept out if suitable services were available" [19].

### The relevance of supporting informal care

Several authors underscore the critical role of the spouse in influencing the living conditions. The presence and the motivation of the co-resident are by far the most essential to enable the provision of home care [9,11,18,20,22].

Support for living conditions, for housing and for the main carer can postpone institutionalisation [17]. The support for informal care is as important as that for direct personal help for the elderly [16,25,26]. For people with Alzheimer's disease, informal care is five times as important as professional care and the people that have to take up this burden are to a large extent the older partners/spouses [27].

The strength or resilience of the informal care is related to intrinsic characteristics. Closeness and a good current relationship between the caregiver and the patient reduce the risk of nursing home placement [28,29].

### Supporting the I-ADL needs is the greatest guarantee to delay or prevent institutionalisation

Trends in nursing home usage suggest that people enter nursing homes at a later age. This may be due to healthy ageing. Older people are living longer and with fewer disabilities [21,30]. However, it may also be due to growth in options [31,32].

A survey of the services for the elderly in Europe illustrates this ever expanding range of types of community care [1]. All sorts of services are becoming increasingly available, enabling older people to live longer independently at home. But feeling in control of the help desired is important for the elderly (self-regulated dependency) [33].

Over the last few years we have seen a noticeable trend towards private entrepreneurs who have discovered the specialised niche market of senior citizens. They are increasingly meeting the wishes of the 'great grey group' in terms of both buildings and the services provided.

Recently, (mostly commercial) 'care hotels' have begun to offer an excellent combination of residence, care and wellbeing. These pure wellness arrangements mainly benefit the wealthiest segment of the ageing population.

### Policy implications

As institutional care is not a primary option for most elderly, home care needs to be stimulated. Supporting the existing main carer for men can lengthen community care. For women, to a large extent widows, a main carer is mostly not available. Sheltered housing and adequate home help can provide the desired increase in care.

In Belgium additional sheltered housing is needed. Almost twenty years after the launch of these 'service houses' only half of the planned capacity has been achieved. The objective was to provide sheltered housing for 2% of people above 60 years. However, this criterion was established at a time when the obvious need for this type of services was less clear than it is today. Indeed, today waiting lists for service houses are much longer than those for nursing homes - five or six years are not unusual. Waiting times for nursing home admission are between 3 and 9 months.

The other type of service that needs enhancing is home help in ordinary housing, which is to a large extent replacing or relieving the main carer. Despite an increase in the last two decades, the capacity of the services nevertheless remains below the required level.

Here again, private providers are increasingly offsetting the shortfall. Besides traditional home help services such as 'meals-on-wheels' and 'cleaning services' offered by the municipalities, the commercial sector is providing more and more supplemental forms of care to complement the services of the municipalities and other non-profit organisations that help with domestic duties and personal care. We are thinking of safety alarms, laundry/ironing services, transport services, home automation and gardening.

In Sweden, private providers delivered about 9% of public care for the elderly in 1999 [32].

## Conclusion

Although different factors seem to affect the request for residential care, our data suggest that the only factor related to an urgent request for nursing home admission is the I-ADL score. Our study provided new evidence confirming the assertion that adequate support for the I-ADL needs of elderly people (in performing housekeeping duties) can avoid unnecessary or premature nursing home admission. The expansion of sheltered housing and the further extension of home help (timely and sufficient professional help and support for the informal carer) could postpone the need for institutionalisation.

## Competing interests

The authors declare that they have no competing interests.

## Authors' contributions

GVR designed the study, collected and analysed the data and wrote the manuscript. JP contributed to writing the manuscript. Both authors read and approved the final manuscript.
